# Examining clustered somatic mutations with SigProfilerClusters

**DOI:** 10.1093/bioinformatics/btac335

**Published:** 2022-05-20

**Authors:** Erik N Bergstrom, Mousumy Kundu, Noura Tbeileh, Ludmil B Alexandrov

**Affiliations:** Department of Cellular and Molecular Medicine, UC San Diego, La Jolla, CA 92093, USA; Department of Bioengineering, UC San Diego, La Jolla, CA 92093, USA; Moores Cancer Center, UC San Diego, La Jolla, CA 92037, USA; Department of Cellular and Molecular Medicine, UC San Diego, La Jolla, CA 92093, USA; Department of Bioengineering, UC San Diego, La Jolla, CA 92093, USA; Moores Cancer Center, UC San Diego, La Jolla, CA 92037, USA; Department of Cellular and Molecular Medicine, UC San Diego, La Jolla, CA 92093, USA; Department of Bioengineering, UC San Diego, La Jolla, CA 92093, USA; Moores Cancer Center, UC San Diego, La Jolla, CA 92037, USA; Department of Cellular and Molecular Medicine, UC San Diego, La Jolla, CA 92093, USA; Department of Bioengineering, UC San Diego, La Jolla, CA 92093, USA; Moores Cancer Center, UC San Diego, La Jolla, CA 92037, USA

## Abstract

**Motivation:**

Clustered mutations are found in the human germline as well as in the genomes of cancer and normal somatic cells. Clustered events can be imprinted by a multitude of mutational processes, and they have been implicated in both cancer evolution and development disorders. Existing tools for identifying clustered mutations have been optimized for a particular subtype of clustered event and, in most cases, relied on a predefined inter-mutational distance (IMD) cutoff combined with a piecewise linear regression analysis.

**Results:**

Here, we present SigProfilerClusters, an automated tool for detecting all types of clustered mutations by calculating a sample-dependent IMD threshold using a simulated background model that takes into account extended sequence context, transcriptional strand asymmetries and regional mutation densities. SigProfilerClusters disentangles all types of clustered events from non-clustered mutations and annotates each clustered event into an established subclass, including the widely used classes of doublet-base substitutions, multi-base substitutions, *omikli* and *kataegis*. SigProfilerClusters outputs non-clustered mutations and clustered events using standard data formats as well as provides multiple visualizations for exploring the distributions and patterns of clustered mutations across the genome.

**Availability and implementation:**

SigProfilerClusters is supported across most operating systems and made freely available at https://github.com/AlexandrovLab/SigProfilerClusters with an extensive documentation located at https://osf.io/qpmzw/wiki/home/.

**Supplementary information:**

Supplementary data are available at *Bioinformatics* online.

## 1 Introduction

Mutations are found on the genomes of all cells in the human body (Martincorena and Campbell, 2015; Stratton *et al.*, 2009). Most single-base substitutions and small insertions and deletions (indels) accumulate independently across the genome, but a subset of the mutations cluster in a non-random manner (Lawrence *et al.*, 2013; Supek and Lehner, 2017). Previous studies have revealed that clustered mutations are imprinted by a plethora of endogenous and exogenous mutational processes (Alexandrov *et al.*, 2020; Boichard *et al.*, 2017; Brash, 2015; Buisson *et al.*, 2019; Chan *et al.*, 2015; Chen *et al.*, 2013; Mas-Ponte and Supek, 2020; Matsuda *et al.*, 1998; Nik-Zainal *et al.*, 2012, 2019; Pfeifer *et al.*, 2005; Roberts *et al.*, 2013, 2012; Supek and Lehner, 2017; Taylor *et al.*, 2013; The ICGC/TCGA Pan-Cancer Analysis of Whole Genomes Consortium, 2020; Wang *et al.*, 2020). Some clustered mutations have been implicated in cancer evolution (Bergstrom *et al.*, 2022; Chen *et al.*, 2013; Mas-Ponte and Supek, 2020; Supek and Lehner, 2017; Taylor *et al.*, 2013; The ICGC/TCGA Pan-Cancer Analysis of Whole Genomes Consortium, 2020), while *de novo* clustered mutations have been identified in the human germline and shown to contribute to developmental disorders (Kaplanis *et al.*, 2019; Veltman and Brunner, 2012). In recent years, sets of simultaneously occurring clustered substitutions have been further subclassified into independent events (Bergstrom *et al.*, 2022; Mas-Ponte and Supek, 2020), including (i) doublet-base substitutions (DBSs); (ii) multi-base substitutions (MBSs); (iii) diffuse hypermutation termed omikli; (iv) longer strand-coordinated events termed kataegis and (v) recurrent hypermutation of extra-chromosomal DNA (ecDNA) termed kyklonas.

Traditional methods separate clustered mutations based on a predefined inter-mutational distance (IMD) threshold typically between 1 and 2 kilobases (Alexandrov *et al.*, 2013, 2020; Chan *et al.*, 2015; D'Antonio *et al.*, 2016; Maciejowski *et al.*, 2020; Nik-Zainal *et al.*, 2019; Taylor *et al.*, 2013). Many of these approaches utilize a piecewise linear regression to segment each chromosome, which, in most cases, is optimized for calling larger strand-coordinated kataegic events (Supplementary Fig. S1) (Alexandrov *et al.*, 2013; Lin *et al.*, 2021; Yin *et al.*, 2020). Most existing methods have also ignored confounding effects attributed to localized differences in mutation rates, copy number alterations or the mutational burden across each chromosome within a given sample leading to an accumulation of false-positive clustered events (Supplementary Fig. S1). Further, the majority of existing tools focus on detecting only a specific class of clustered events including doublet-base substitutions and multi-nucleotide variants (Chen *et al.*, 2013; Matsuda *et al.*, 1998; Wang *et al.*, 2020), kataegis (D'Antonio *et al.*, 2016; Lin *et al.*, 2021; Taylor *et al.*, 2013) or APOBEC3-associated events (Chan *et al.*, 2015; Nik-Zainal *et al.*, 2012) while ignoring the larger landscape of clustered mutations. For example, a recent study (Mas-Ponte and Supek, 2020) developed an algorithm focused on the detection of APOBEC3-associated omikli and kataegis events in cancer genomes by incorporating simulations of somatic mutations and estimates of cancer cell fractions.

**Fig. 1.**
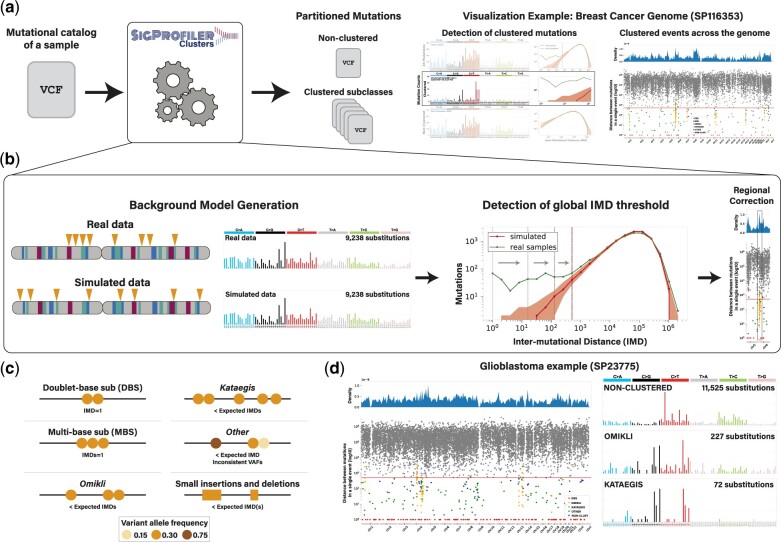
Detection and characterization of clustered mutations with SigProfilerClusters. (**a**) An example workflow used to detect clustered mutations in a single cancer genome. As an input, SigProfilerClusters accepts common formats for mutations, such as ones in the variant calling format (VCF), and the tool separates all clustered mutations from the complete mutational catalog of the provided sample. Final partitions of mutations in the sample are outputted as VCF files and visualized using the mutational spectra of all mutations, only clustered mutations and only non-clustered mutations along with a rainfall plot commonly used to show the distribution of inter-mutational distances across a cancer genome (Alexandrov *et al.*, 2013; Bergstrom *et al.*, 2022; Nik-Zainal *et al.*, 2012). (**b**) Schematic demonstrating the process of calculating a sample-dependent IMD threshold to separate clustered from non-clustered mutations across each genome. A binary search algorithm is used to efficiently detect the optimal global IMD threshold for each sample. Detection of the global IMD threshold is illustrated using gray arrows. Regional corrections are performed to identify local IMD thresholds based on variance of mutation rates across the genome. (**c**) Every clustered mutation is classified into a single subcategory of clustered event. (**d**) Rainfall plot illustrating the distribution of IMDs across a single glioblastoma sample (*left*). The mutational spectra for *omikli* and *kataegic* events reveal a different mutational pattern compared to the pattern of all non-clustered somatic mutations (*right*)

Separation and classification of clustered events are required to fully elucidate the mutational processes operating in cancer and normal somatic cells (Bergstrom *et al.*, 2022; Supek and Lehner, 2017). Here, we present SigProfilerClusters, a tool to comprehensively characterize and subclassify clustered mutations from the complete catalog of mutations within the genome of a single sample (Fig. 1a). SigProfilerClusters classifies all types of clustered mutations, including (i) doublet-base substitutions; (ii) multi-base substitutions; (iii) omikli; (iv) kataegis and (v) clustered small insertions and deletions (indels). The tool calculates a sample-dependent IMD threshold that considers regional differences in mutation rates, variant allele fractions and cancer cell fractions of adjacent mutations to reduce the false positive rate and provides visualizations for downstream analyses (Fig. 1b and c; Supplementary Fig. S1). Further, SigProfilerClusters integrates within the larger suite of SigProfiler tools (Bergstrom *et al.*, 2019, 2020; Islam *et al.*, 2020) to facilitate downstream mutational signature analysis of both non-clustered and clustered single-base substitutions and indels, thus, allowing the accurate detection of mutational processes giving rise to even low levels of clustered events (Fig. 1d) (Bergstrom *et al.*, 2019, 2022; Islam *et al.*, 2020).

## 2 Materials and methods

SigProfilerClusters derives an IMD cutoff that is unlikely to occur purely by chance given the observed mutational burden and the mutational patterns within the genome of a given sample. To calculate the genome-dependent IMD, the tool leverages SigProfilerSimulator (Bergstrom *et al.*, 2020) to generate background models by randomizing the distribution of mutations across the genome. By default, the genome of each sample is simulated 100 times in order to derive 95% confidence intervals for the expected genomic mutational landscape, with every simulation maintaining the penta-nucleotide sequence context for each substitution, the ratio of all mutations in genic and inter-genic regions, the transcriptional strand asymmetries of all mutations in genic regions and the mutational burden on each chromosome (Bergstrom *et al.*, 2019, 2020). Importantly, this randomization procedure is highly customizable (Bergstrom *et al.*, 2020) and can be altered based on the needs of a given study design, thus, allowing the incorporation of other factors that affect the accumulation of mutations such as nucleosome occupancy, presence of histone modifications and many others. A binary search algorithm is implemented to efficiently derive the global IMD threshold for each genome. The final global IMD threshold is selected by ensuring that 90% of mutations below the chosen cutoff are unlikely to appear by chance given the simulated distribution of mutations (q-value < 0.01; Supplementary Fig. S1) with a maximum global IMD cutoff of 10 kilobases. The algorithm also considers regional heterogeneities of mutation rates, generally associated with replication timing (Stamatoyannopoulos *et al.*, 2009) or differential gene expression (Buisson *et al.*, 2019; Hess *et al.*, 2019; Lawrence *et al.*, 2013; Pleasance *et al.*, 2010; Polak *et al.*, 2015), by correcting for variance in clonality as well as variance in both mutation-dense and mutation-poor regions using a sliding genomic window (default size of 1 megabase). Specifically, an additional regional IMD cutoff is corrected within each genomic window based on the fold difference between the number of real and the number of simulated mutations, while maintaining the original criteria of <10% of mutations below the IMD cutoff appearing by chance (q-value < 0.01). Lastly, when data are available, SigProfilerClusters ensures that adjacent mutations are in the same cells by introducing a maximum difference in variant allele frequencies (VAF) or cancer cell fraction (CCF), which incorporates copy number changes, below a certain threshold (default cutoff value of 0.10 and 0.25; respectively).

After identifying the set of clustered mutations, SigProfilerClusters subclassifies each clustered substitution into a single category of previously established clustered events (Bergstrom *et al.*, 2022; Mas-Ponte and Supek, 2020). Briefly, all clustered substitutions with consistent VAFs or consistent CCFs are classified into one of four categories. Two mutations with an IMD of 1 are classified as doublet-base substitutions, while clusters of three or more adjacent mutations each with an IMD of 1 are classified as multi-base substitutions. Clusters of two or three mutations with IMDs less than the sample-dependent cutoff and with at least a single IMD greater than 1 are classified as omikli (Bergstrom *et al.*, 2022), while clusters of four or more mutations with IMDs less than the sample-dependent cutoff and with at least a single IMD greater than 1 are classified as kataegis (Bergstrom *et al.*, 2022). All remaining clustered mutations with inconsistent VAFs or CCFs are classified as other. Clustered indels are not subclassified into different categories due to a lack of previously defined subtypes.

## 3 Usage

SigProfilerClusters is freely available as a Python package, distributed under the permissive BSD-2 clause license and can be used on most operating systems including Windows, MacOS and Linux-based machines. The tool is compatible with large-scale deployments on high-performance computing clusters as well as on cloud infrastructures such as Amazon Web Services. Input data can be provided in the form of common mutation formats including the Variant Call Format (VCF), the Mutation Annotation Format or in the form of a simple text file. The output of SigProfilerClusters results in the partitioning of all mutations into a clustered or non-clustered directory. All clustered mutations are then classified into distinct subcategories of events and provided individually in VCF files for downstream visualization and analyses. The output for each subclass of the clustered event can be directly utilized by additional SigProfiler tools including SigProfilerExtractor for mutational signature analysis (Islam *et al.*, 2020) and SigProfilerPlotting for examining patterns of somatic mutations (Bergstrom *et al.*, 2019). The results for each sample are also summarized using two individual visualizations that include: (i) a rainfall plot depicting the minimum global IMD between all adjacent mutations, where each individual set of adjacent mutations is colored based on its clustered classification; and (ii) a multi-panel figure that displays the mutational patterns across all mutations, clustered mutations and non-clustered mutations, separately along with the distribution of IMDs across the real and simulated data for each sample (Fig. 1a).

## 4 Conclusion

Elucidating the compendium of clustered somatic mutations in the genome of a sample allows further understanding of the mutational process that give rises to these events and can provide novel insights into disease etiology (Bergstrom *et al.*, 2022; Mas-Ponte and Supek, 2020; Supek and Lehner, 2017). Previous studies have traditionally interrogated the complete mutational catalogs of cancer genomes, which can lead to the inability to detect processes active at low levels or those which have been transiently activated. Our prior analysis of clustered mutations (Bergstrom *et al.*, 2022) has revealed an enrichment of clustered mutations within known cancer driver events, hypermutation of extra-chromosomal DNA fueling the evolution of cancers, and ultimately, resulting in a differential patient outcome. Here, we provide SigProfilerClusters, an automated and freely available Python-based tool that comprehensively identifies and classifies clustered mutations enabling users to interrogate the mutational processes giving rise to such events.

## Author contributions

E.N.B. developed the Python code and wrote the manuscript. M.K. performed all benchmarking. E.N.B., M.K. and N.T. tested and documented the code. L.B.A. supervised the overall development of the code, benchmarking and writing of the manuscript. All authors read and approved the final manuscript.

## Funding

This was work was supported by Cancer Research UK Grand Challenge Award [C98/A24032] as well as US National Institute of Health [R01ES030993-01A1 and R01ES032547]; a Packard Fellowship for Science and Engineering to L.B.A. The funders had no roles in study design, data collection and analysis, decision to publish or preparation of the manuscript.

*Conflict of Interest*: L.B.A. is a compensated consultant and has equity interest in io9, LLC. His spouse is an employee of Biotheranostics, Inc. L.B.A. is also an inventor of a US Patent 10,776,718 for source identification by non-negative matrix factorization. ENB and LBA declare provisional patent applications for ‘Clustered mutations for the treatment of cancer’ (U.S. provisional application serial number 63/289,601) and ‘Artificial intelligence architecture for predicting cancer biomarker’ (serial number 63/269,033). All other authors declare no competing interests.

## Data Availability

No data were generated for this publication.

## Supplementary Material

btac335_Supplementary_Data
