# Supplementary figures and images for: Examining clustered somatic mutations with SigProfilerClusters
Source: Bioinformatics. 2022 May 20;38(13):3470–3. doi: 10.1093/bioinformatics/btac335 (PMC9237733; doi:10.1093/bioinformatics/btac335)

**Supplementary Figure 1. Benchmarking of existing tools that detect clustered mutations**

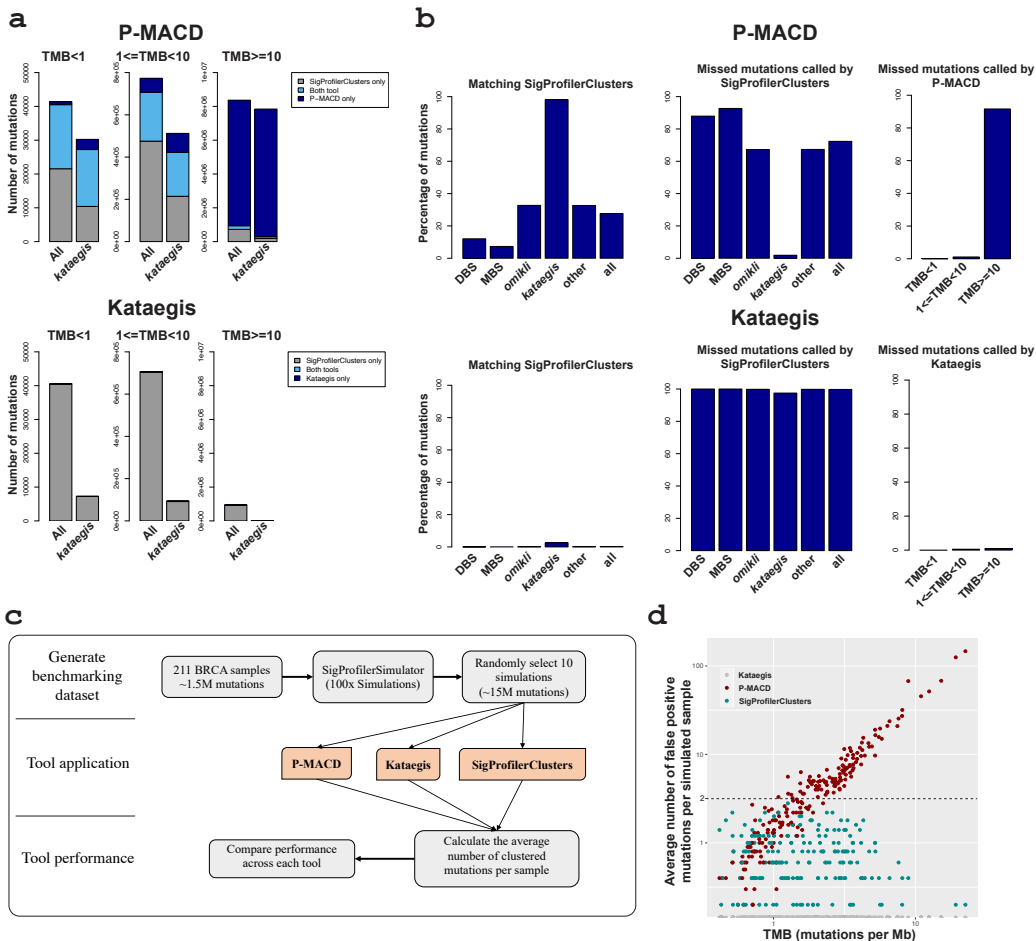

Supplement: btac335_Supplementary_Data [file btac335_supplementary_data.pdf]
